# Comprehensive evaluation of health status of Tibetan primary and middle school students based on an artificial neural network model: A cross-sectional study

**DOI:** 10.1097/MD.0000000000050076

**Published:** 2026-08-14

**Authors:** Chenyao Mai, Xiaofei Liu, Qiuyu Pan, Rulin Li, Pengcheng Zhang, Jun Hu

**Affiliations:** a College of Medical, Xizang University of Medicine, Lasa, China; b Key Laboratory of Digital-Intelligent Disease Surveillance and Health Governance, North Sichuan Medical College, Nanchong, China; c School of Music, Shandong Women’s University, Jinan, Shandong, China; d College of Public Health, North Sicuan Medical College, Nanchong, China; e College of Health Management, Shandong University of Traditional Chinese Medicine, Jinan, China.

**Keywords:** artificial neural network, health evaluation, primary and middle school students, Tibet

## Abstract

The high-altitude and unique climatic conditions of Tibet can have a significant impact on the growth and development of local children and adolescents. We aimed to apply an artificial neural network model to evaluate the physical health status of primary and secondary school students aged 7 to 18 years in Shigatse, Tibet, China. A cross-sectional study was conducted among eligible primary and secondary school students from 7 randomly selected schools in Shigatse, Tibet, China, between April and October 2024. Health-related data, including height, weight, lung vital capacity, visual acuity, and dental caries, were recorded. By applying a comprehensive evaluation index system, an artificial neural network model was constructed to evaluate the health status of these students. A total of 4106 students (2047 boys and 2059 girls) were included. The body shape score (based on body mass index), body mass index of vital capacity, and health disorder score were 72.83 ± 12.29, 70.97 ± 8.45, and 69.26 ± 9.52, respectively. The corresponding measurements for boys and girls were 72.58 ± 12.22 and 73.07 ± 12.37, 69.02 ± 8.33 and 72.91 ± 8.11, and 69.22 ± 9.69 and 69.30 ± 9.36, respectively. The prevalence of myopia, dental caries, severe underweight, underweight, overweight, obesity, and growth retardation was 59.24%, 70.21%, 8.04%, 7.84%, 3.97%, 1.14%, and 7.67%, respectively. The mean and variance of health comprehensive scores were 70.42 ± 5.88, with 70.06 ± 5.75 and 70.77 ± 5.99 for boys and girls, respectively. The overall health status of Tibetan primary and secondary school students was moderate. Underweight, dental caries, and myopia were important health problems in these students. Public health departments, schools, and parents should work together to provide more comprehensive health education, supply necessary resources, and guide these students in adopting a healthy lifestyle to promote normal growth and development.

## 1. Introduction

Childhood and adolescence are critical for growth and development. Their physical status is related not only to individuals’ healthy growth and happy lives, but also impacts the health of the entire nation in the future.^[1]^ Healthy young people are the foundation of the Chinese Dream.^[2]^ The “Healthy China 2030” plan outlines specific strategies for building a healthy China and highlights the health problems of key populations, including children and adolescents. Understanding their health status can help tailor policymaking and resource utilization.

In Tibet, an autonomous region of China, the high-altitude plateau, its special natural environment, and climatic conditions, along with the local population’s socioeconomic background, pose a significant health challenge. In addition, the constant year-round low temperature and lack of oxygen lead Tibetan people to experience a wide range of physiological and genetic changes, resulting in unique physical health characteristics. Tibetan children and adolescents commonly have lower height and weight than the national averages for the same age groups. Although a small proportion of children may be obese, a large number often have malnutrition and growth retardation, particularly in remote rural areas of Tibet.^[3]^ According to a 2014 report on the physical fitness and health surveillance of Chinese school students, the myopia rate was 56.67% in boys and 69.19% in girls among Tibetan primary and secondary school students.^[4]^ In addition, the prevalence of dental caries was high, and awareness of oral healthcare and the desire for treatment were low.^[5]^ Here, we conducted the present study to further explore the impact of the natural environment on the health status of primary and secondary school students in Tibet.

## 2. Materials and methods

### 2.1. Study design and participants

From April to October 2024, we conducted a cross-sectional study of students from 7 primary and secondary schools randomly selected from Sangzizhu District, Bainang County, and Rinbung County in Shigatse, Tibet, China. These 3 mountainous areas are located in southwestern Tibet, with an average altitude of more than 4000 meters. Most of the students were from agricultural and pastoral families. The study protocol was approved by the ethics committees of the local government education department and each participating school. Students aged 7 to 18 years in these 7 schools were invited to participate in the study. Their parents provided informed consent.

### 2.2. Health status examinations

The health status examinations included height, weight, lung vital capacity, naked-eye visual acuity, and dental caries. Measurements followed the guidelines of the national student physical health survey. Research personnel received training to use uniform testing methods and equipment.^[6]^ Height and weight were recorded to 0.1 cm and 0.1 kg, respectively. Lung vital capacity was measured twice with an electronic spirometer at intervals of no more than 15 seconds, and the maximum value was recorded. The vision test was performed by ophthalmologists, and the results were recorded using a 5-point scoring system with a reference range of 3.3 to 6.0. Dental professionals performed dental caries examinations in accordance with the diagnostic criteria published by the World Health Organization.^[7]^ The number of carious lesions was determined and recorded.

### 2.3. Statistical analysis

After reviewing the relevant literature and theories, we applied a comprehensive evaluation index system compiled by Wu et al^[8–10]^ to analyze the health status of primary and secondary school students in Tibet. This index system comprises 3 categories of indicators: body shape (body mass index [BMI]), physiological function (BMI of vital capacity), and health disorders (poor vision, dental caries, obesity, overweight, underweight, and growth retardation).^[11,12]^ The weight coefficients were 0.25, 0.25, and 0.5 for BMI, physiological function, and health disorders, respectively (Tables 1 and 2).^[8]^ Using the percentiles of Han male and female students in the 2019 Report on the Physical Fitness and Health Surveillance of Chinese School Students as cutoff values, the measurements in the current study were categorized into 5 levels: upper, upper-middle, middle, lower-middle, and lower, which corresponded to standard scores of 90, 80, 70, 60, and 50, respectively. The standard score of each index was multiplied by the corresponding weight coefficient to obtain the weighted standard score of each index. According to the comprehensive evaluation framework proposed in previous studies ^[10,13,14]^, health indicators were classified into 5 levels, including upper, upper-middle, middle, lower-middle, and lower, corresponding to standard scores of 90, 80, 70, 60, and 50, respectively. The comprehensive health score was obtained by summing up the weighted standard scores of the 3 dimensions: body shape, physiological function, and health disorders. The formula for calculating the composite index score is $P=\sum w_{i}M_{i}\left(i=1,2,3\right)$, where P is the comprehensive index score, $W_{i}$ is the weight of each index, and $M_{i}$ is the standard score of each index.

**Table 1:** Definition of body shape, physiological function, and health disorders.^[8,10]^

Index	Content	Weight coefficient	Grading criteria
Body shape	BMI	0.25	Upper level (90): $P_{50}\leq \;$ BMI $<P_{70}$
			Upper-middle level (80): $P_{30}\leq$ BMI $<P_{50}$
			Middle level (70): $P_{15}\leq$ BMI $<P_{30}$ or $P_{70}\leq \;$ BMI $<P_{85}$
			Lower-middle level (60) $:\;P_{5}\leq \;$ BMI $<P_{15}\;or\;P_{85}\leq$ BMI $<P_{95}$
			Lower level (50): BMI $<P_{5}\;$ or BMI $\geq P_{95}$
Physiological Function	Body mass index of vital capacity	0.25	Upper level (90): vital capacity/BMI $\geq P_{97}$
			Upper-middle Level (80): $P_{75}\leq$ vital capacity/BMI $<P_{97}$
			Middle level (70): $P_{25}\leq$ vital capacity/BMI $<P_{75}$
			Lower-middle level (60): $P_{3}\leq$ vital capacity/BMI $<P_{25}$
			Lower level (50): vital capacity/BMI $<P_{3}$
Health disorders	Poor vision	0.5	Upper level (90): 100
	Dental caries		Upper-middle level (80): 97–99
	Obesity or overweight		Middle level (70): 87–96
	Underweight		Lower-middle Level (60): 80–86
	Organic diseases		Lower level (50):<80

BMI = body mass index.

**Table 2:** Definition and scoring system for health disorders.

Health disorder	Criteria	Points assigned
Myopia (vision <5.0)	Mild: visual acuity = 4.0	Mild 3, moderate 4, severe 10
	Moderate: 4.6 ≤visual acuity ≤4.8	
	Severe: visual acuity ≤4.5	
Dental caries	The lesions on the pit and fissure or smooth surface of the teeth include softening of the base, potential damage to the enamel, or muralization	2 Points per tooth, Max. 10
Obesity or overweight	Body mass index values for screening of overweight and obesity among school-aged children and adolescents in China^[11]^	Overweight:10, obesity:10
Underweight	Screening for malnutrition among school-aged children and adolescents in China^[12]^	Growth retardation: 5, mild underweight: 5, and severe underweight: 10
Organic diseases	Diagnosis report of secondary and tertiary hospitals	One disease: 30

Physical Health Score is the total score of 100 points minus the sum of the scores of different diseases.^[13]^

Statistical analyses were performed in SPSS software (version 26.0, IBM Corp., Armonk) and MATLAB (R2021B, MathWorks). Continuous variables were expressed as mean ± standard deviation. Categorical variables were reported as frequencies and percentages. Normality distribution of continuous variables was assessed using the Shapiro–Wilk test and histograms. Continuous variables with approximate normal distributions were compared using independent-samples *t* tests, while continuous variables without normal distributions were compared with Mann–Whitney Test. Categorical variables were analyzed using Chi-square test or Fisher exact test. All tests were two-sided, with a *P* < .05 considered statistically significant.

An artificial neural network (ANN) model was constructed using MATLAB to evaluate the comprehensive health status of students. The input variables included height, weight, vital capacity, left and right visual acuity, dental caries status, and altitude-related factors. ANN model was used because the health evaluation framework involved multiple physiological and clinical indicators that might interact in complex nonlinear ways.^[15]^ Traditional statistical models such as logistic regression assume linear relationships and may not fully capture these multidimensional associations. Therefore, a multilayer perceptron neural network was employed to generate a comprehensive health evaluation score.

Because the plateau environment affects the development of children’s height, weight, and lung vital capacity, the physical development of children and adolescents in plateau areas generally lags behind that of students in plain areas of the same age, resulting in a lower BMI at high-altitude than at low-altitude.^[16]^ The special natural conditions in plateau areas (such as low air pressure, low oxygen, and extreme cold) have a significant impact on growth and development, resulting in shorter height and lower weight.^[17]^ These changes are mainly due to physiological adaptation rather than pathological abnormalities. However, the current “student physical health standard” used in China is based primarily on data from Han students in plain areas. Directly applying this standard to students living at high-altitudes can easily lead to evaluation distortion and an increased risk of misjudgment. Therefore, we constructed an “equivalent age + altitude correction” model to improve accuracy by accounting for altitude-related developmental delay (about 0.25 years per 1000 m increase).^[18]^ For each 1000 m increase in elevation, children’s height-for-age Z-scores decreased by an average of approximately 0.195–0.2, equivalent to a developmental age lag of approximately 0.2 to 0.3 years.^[19]^ The correction formula of equivalent age is $\Delta A=k(\frac{E}{1000})$, $Age_{equiv}=A-\Delta A$), where A is the actual age of the student (years), E is the altitude of the place of residence (in meters), and k is the influence factor of altitude on growth age, taking the empirical median of 0.25.

## 3. Results

### 3.1. General information about primary and middle school students

A total of 4106 Tibetan primary and secondary school students were included in this study, of whom 49.85% (2047) were boys and 50.15% (2059) were girls. The proportions of primary school, junior high school, and high school students were 26.9%, 50% and 23%, respectively. The distribution of students across age groups from 7 to 18 years was as follows: 2.14% (88), 3.97% (163), 5.23% (215), 5.21% (214), 4.94% (203), 4.14%(170), 7.05% (290), 15.39% (633), 17.60% (724), 12.76% (525), 11.88% (489), and 9.52% (392), respectively.

### 3.2. Body shape (BMI) measurement

**Figure 1.**
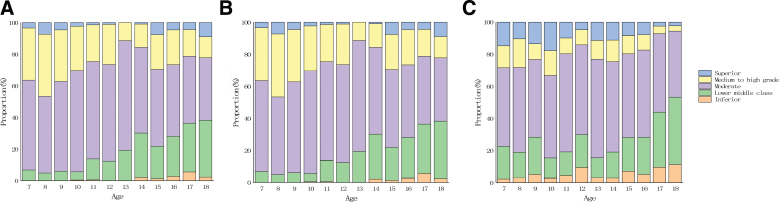
Distributions of body shape (A), physiological function (B), and health disorders (C) into different categories in different age groups.

The average height of girls was higher than that of boys in the 8 to 13-year age group, whereas the average height of boys exceeded that of girls after age 14. Boys weighed more than girls before age 12, girls weighed more than boys between ages 12 and 14, and boys again weighed more than girls after age 14. The BMI was 18.38 ± 2.74 (Table 3), with 18.25 ± 2.60 for boys and 18.51 ± 2.87 for girls. The BMI of boys was significantly higher than that of girls in the 7- to 12-year age group, whereas the BMI of girls was higher than that of boys after age 12. In addition, the BMI difference first increased with age, then decreased, and increased again, with a significant difference between boys and girls (*P* < .05). The average standard score for body shape was 72.83 ± 12.29 in primary school students (Table 3), with 72.58 ± 12.22 in boys and 73.07 ± 12.37 in girls. The numbers and frequencies of body shape scores in the upper, upper-middle, middle, lower-middle, and lower levels were 409 (9.96%), 658 (16.03%), 1203 (29.30%), 1035 (25.21%), and 801 (19.51%), respectively (Fig. 1A). There was no significant difference in the body shape distribution between sexes (*P* ≥ .05).

**Table 3:** Body mass index and body shape score of primary and secondary school students by sex at different ages.

Age, yr	Body mass index (M ± SD)		Body shape score (M ± SD)	
	Boys	Girls	Boys	Girls
7	15.28 ± 2.45	14.61 ± 1.47	70.87 ± 14.88	70.00 ± 13.79
8	15.35 ± 2.36	14.45 ± 2.02	70.49 ± 14.40	66.59 ± 14.07
9	15.76 ± 2.50	14.99 ± 2.19	68.56 ± 13.07	68.64 ± 13.00
10	16.62 ± 2.44	16.07 ± 2.89	74.91 ± 12.21	75.19 ± 12.34
11	17.58 ± 2.37	17.07 ± 2.04	76.29 ± 12.53	76.23 ± 10.73
12	18.82 ± 3.51	18.19 ± 2.56	73.42 ± 12.04	73.61 ± 10.82
13	18.99 ± 2.10	19.33 ± 2.14	80.00 ± 8.63	77.43 ± 9.95
14	18.73 ± 1.98	18.78 ± 1.90	76.91 ± 10.15	75.07 ± 11.14
15	18.31 ± 2.43	18.99 ± 2.55	70.46 ± 12.16	70.77 ± 12.35
16	18.29 ± 2.09	19.3 ± 3.08	69.53 ± 12.40	71.14 ± 12.96
17	19.51 ± 2.63	20.23 ± 2.61	70.96 ± 11.47	74.33 ± 12.38
18	19.46 ± 1.99	20.51 ± 2.34	70.00 ± 11.30	74.27 ± 12.38

M ± SD = mean ± standard deviation.

### 3.3. Physiological function (BMI of vital capacity) measurement

The lung vital capacity of Tibetan primary and secondary school students was 2263.56 ± 749.13, with 2377.89 ± 789.31 and 2149.90 ± 688.54 for boys and girls, respectively. The BMI of vital capacity was 52.04 ± 12.92, with 53.76 ± 13.35 and 50.34 ± 12.24 for boys and girls, respectively (Table 4). There was a significant difference in the BMI of vital capacity between sexes (*P* < .05). With increasing age, the vital capacity of the BMI increased gradually. The vital capacity and BMI of boys were significantly higher than those of girls at all ages. However, after age 12, the difference in vital capacity between boys and girls increased with age, and the average vital capacity of boys and girls in high-altitude areas was lower than that in low-altitude areas. The standard score of the BMI of vital capacity was 70.97 ± 8.45, with 69.02 ± 8.33 and 72.91 ± 8.11 for boys and girls, respectively (Table 4). The numbers and frequencies of physiological function levels in the upper, upper-middle, middle, lower-middle, and lower categories were 74 (1.80%), 894 (21.77%), 2113 (51.46%), 848 (20.65%), and 177 (4.31%), respectively (Fig. 1B). There was a significant difference in the distribution of physiological function levels between boys and girls (*P* < .05).

**Table 4:** Body mass index of vital capacity of primary and middle school students by sex at different ages.

Age, yr	Body mass index of vital capacity (M ± SD)		Physiological function (M ± SD)	
	Boys	Girls	Boys	Girls
7	48.71 ± 13.16	52.16 ± 10.41	73.83 ± 6.99	74.52 ± 5.93
8	52.86 ± 12.31	55.95 ± 12.26	72.17 ± 6.96	75.98 ± 7.00
9	55.76 ± 13.95	53.25 ± 9.35	74.12 ± 7.04	73.14 ± 6.37
10	54.79 ± 10.50	51.42 ± 9.68	72.83 ± 6.29	72.5 ± 6.29
11	52.84 ± 11.59	50.26 ± 9.68	71.03 ± 6.84	71.32 ± 6.84
12	53.66 ± 9.85	51.17 ± 9.30	71.23 ± 6.44	71.75 ± 6.44
13	49.34 ± 8.68	47.82 ± 7.71	68.22 ± 5.59	70.21 ± 5.59
14	49.6 ± 13.20	47.54 ± 9.21	66.88 ± 7.43	70.1 ± 7.43
15	56.44 ± 15.08	52.08 ± 13.25	69.78 ± 8.84	73.12 ± 8.84
16	57.05 ± 13.73	50.28 ± 13.40	68.86 ± 8.35	71.62 ± 8.34
17	52.69 ± 12.66	48.98 ± 14.46	65.43 ± 8.08	70.78 ± 8.08
18	55.76 ± 13.65	49.82 ± 15.83	67.26 ± 8.42	70.68 ± 8.42

M ± SD = mean ± standard deviation.

### 3.4. Health disorder measurement

The prevalence of myopia was 59.24%, with prevalences of 37.79%, 60.42% and 81.82% in primary school, junior high school, and senior high school, respectively. The prevalence of myopia among 13–14-year-olds was higher than that in other age groups. The prevalence in girls was significantly higher than that in boys (*P* < .001). The frequency of dental caries was 70.21%. In terms of age distribution, 11- to 12-year-old students had a higher rate of dental caries, and age 18 was the peak for dental caries. The prevalence of dental caries in primary school, junior high school, and senior high school was 71.34%, 66.02%, and 78.01%, respectively.

The prevalences of overweight and obesity were 3.97% and 1.14%, respectively; overweight was 4.69% in boys and 3.25% in girls, and obesity was 1.27% in boys and 1.02% in girls. The prevalence of obesity was 8.77%, 2.92%, and 5.60% in primary school, junior high school, and senior high school, respectively. The prevalence of growth retardation was 7.67%, with 10.16% in boys and 5.20% in girls. The prevalence of growth retardation in primary school, junior high school, and senior high school was 12.39%, 8.62% and 0.11%, respectively. The prevalences of moderate and severe underweight were 7.84% and 8.04%, respectively.

The prevalences of mild and moderate-to-severe underweight were 10.41% and 5.29% in boys, and 9.23% and 6.85% in girls, respectively. The prevalence of mild, moderate, and severe underweight in boys was significantly higher than in girls (*P* < .05). The prevalences of underweight were 17.09%, 16.80%, and 12.47% in primary school, junior high school, and senior high school, respectively. The prevalence of growth retardation was 12.39%, 8.62%, and 0.11% in primary school, junior high school, and senior high school (Table 5). Students scored 69.26 ± 9.52 on the health disorders score, with 69.22 ± 9.69 for boys and 69.30 ± 9.36 for girls (Table 5). The numbers and frequencies of health disorders were 247 (6.00%), 932 (22.70%), 2157 (52.53%), 418 (10.18%), and 352 (8.57%) in the upper, upper-middle, middle, lower-middle, and lower levels, respectively (Fig. 1C). There was no significant difference in the distribution of health disorder levels between boys and girls.

**Table 5:** Disease prevalence and health disorders among primary and middle school students at different ages.

Age	Myopia (%)			Obesity (%)		Underweight (%)		Growth retardation (%)	Dental caries (%)	Health disorders (M ± SD)	
	Mild	Moderate	Severe	Overweight	Obesity	Mild	Severe			Boys	Girls
7	19.32	13.64	1.14	3.41	3.41	7.95	15.91	4.55	63.64	71.36 ± 9.19	74.29 ± 9.41
8	17.79	7.98	1.84	3.07	3.07	3.68	23.93	13.50	66.87	69.57 ± 10.10	71.95 ± 9.35
9	14.88	16.28	7.91	2.33	5.12	7.91	18.14	6.98	66.98	69.48 ± 10.55	71.02 ± 10.24
10	10.75	14.02	6.54	1.40	4.21	6.07	7.01	11.68	67.29	72.55 ± 10.61	73.98 ± 9.37
11	8.87	19.70	5.91	1.48	6.90	5.91	2.96	16.75	79.80	70.41 ± 10.10	70.75 ± 8.01
12	12.94	35.29	7.65	4.12	11.76	8.24	1.18	12.35	79.41	67.12 ± 8.74	68.56 ± 9.35
13	21.72	36.90	4.83	1.38	4.48	1.03	0.69	5.52	61.03	71.71 ± 8.97	71.46 ± 8.93
14	16.59	29.70	17.06	0.16	3.00	9.16	2.21	3.16	63.19	71.38 ± 9.25	71.37 ± 9.17
15	10.91	24.86	22.38	1.10	2.07	8.84	11.88	14.92	69.89	69.81 ± 9.64	68.69 ± 9.78
16	12.19	23.81	25.52	0.19	2.29	11.43	13.90	8.19	66.67	68.55 ± 8.94	69.96 ± 9.41
17	6.95	19.84	53.78	1.02	5.93	5.93	5.32	1.23	77.91	65.8 ± 9.32	65.44 ± 7.29
18	6.38	14.80	60.20	0.51	3.32	9.95	3.57	0.26	81.38	64.52 ± 9.01	64.17 ± 7.46

M ± SD = mean ± standard deviation.

### 3.5. Comprehensive evaluation of health status by artificial neural network

**Figure 2.**
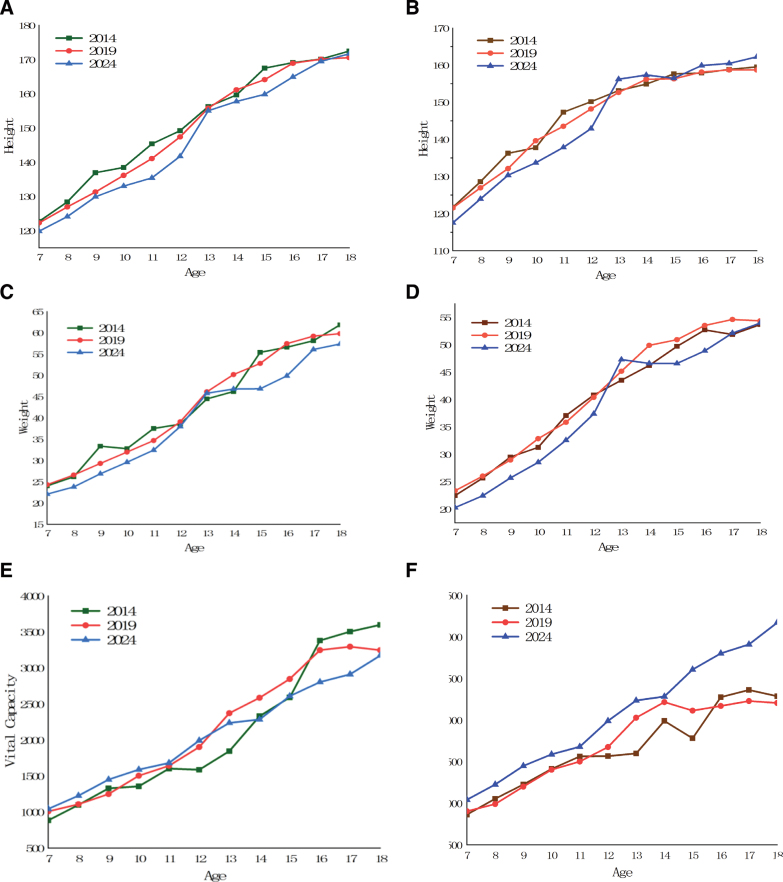
Receiver operator characteristic curve of artificial neural network.

In this study, a 3-layer neural network model was created in MATLAB, 6 original variables from body shape, physiological function, and health disorder measurements (height, weight, vital capacity, left eye visual acuity, right eye visual acuity, and dental caries), plus the altitude factor, were used as the independent variables, and health status was the dependent variable. According to the size of the dataset and the literature, we defined two hidden layers in the model containing 15 and 10 neurons, respectively, and set the maximum number of training iterations to 500.^[20–22]^ The data were divided into training, test, and validation sets in a 7: 1.5: 1.5 ratio. After the 31st training round, the gradient began to stabilize, and the training set fit reached its maximum (*R* = 0.91025). The fitting effects for the validation and test sets were slightly lower (*R* = 0.8815 and 0.85601, respectively. The overall dataset fit was satisfactory (*R* = 0.89821), and model performance Mae = 2.19) indicated strong generalization ability. ROC curve analysis yielded an area under the curve of 0.93993, indicating high predictive accuracy ^[23]^, strong judgment ability, and a relatively low probability of misjudgment (Fig. 2).

**Figure 3.**
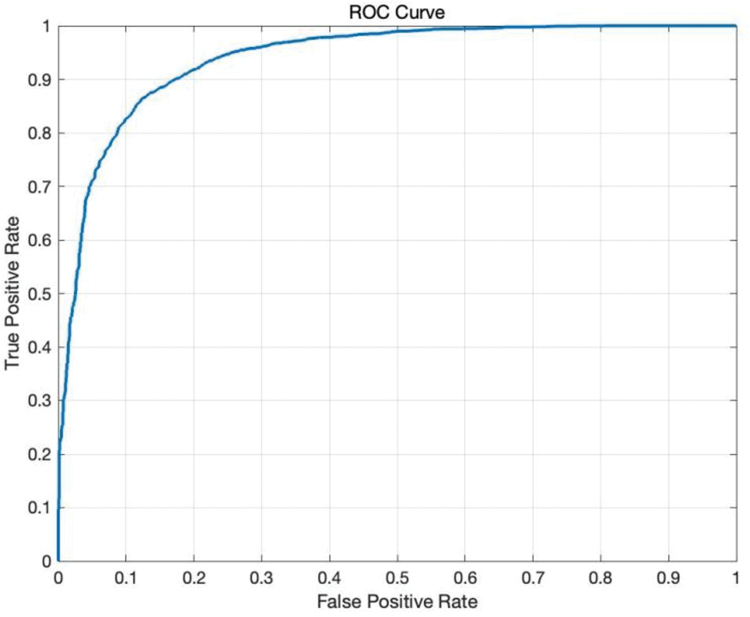
Trend of the scores of the health comprehensive evaluation with age.

The comprehensive health score was 70.42 ± 5.88, with 70.06 ± 5.75 for boys and 70.77 ± 5.99 for girls (Table 6). The comprehensive health score reached its highest value at age 10. With increasing age, the comprehensive health score decreased. By sex, girls had the highest score at age 7 and the lowest at age 18, while boys had the highest score at age 10 and the lowest at age 17 (Fig. 3).

**Table 6:** Comprehensive health evaluation scores of primary and middle school students by sex at different ages.

Age, yr	Boys (M ± SD)	Girls (M ± SD)
7	68.73 ± 5.89	68.56 ± 4.58
8	70.38 ± 5.16	68.84 ± 5.66
9	69.99 ± 6.71	69.14 ± 6.13
10	68.36 ± 4.81	70.25 ± 5.68
11	71.35 ± 5.45	70.73 ± 5.11
12	70.24 ± 5.79	72.55 ± 5.51
13	70.2 ± 5.43	70.62 ± 6.22
14	71.67 ± 4.88	72.48 ± 5.01
15	69.42 ± 6.10	68.37 ± 5.50
16	67.62 ± 5.17	71.17 ± 6.24
17	71.18 ± 5.90	73.07 ± 6.19
18	71.48 ± 5.94	70.54 ± 6.35

M ± SD = mean ± standard deviation.

## 4. Discussion

In the present study, the mean comprehensive health score of Tibetan primary and secondary school students was 70.42 ± 5.88. According to the evaluation criteria proposed by Zhang Y et al^[14]^, this score corresponded to the middle level, indicating that the overall health status of this population was moderate. The comprehensive health scores of boys ages 8 to 11 and 13 to 15 were higher than those of girls. Most students fell into the middle categories of body shape, physiological function, and health disorders. The identified health issues were similar to previous findings, mainly including dental caries, myopia, underweight, overweight, and obesity. The most prominent problems were overweight, obesity, and growth retardation in primary school students, and myopia and dental caries in high school students.

According to the China Child Development Report (2021),^[24]^ approximately 67.9% of primary and secondary school students in China had myopia in 2019. The prevalence of myopia among Tibetan primary and secondary school students was 59.3%, which was slightly lower than the national average and within the range reported in previous studies.^[25]^ The relatively lower prevalence compared with some urban regions might be associated with increased outdoor exposure and lower levels of digital device use in high-altitude rural environments. However, our study showed that a significant number of Tibetan students (59.3%) still faced vision health problems, which may be attributed to factors such as prolonged study hours and the use of electronic devices. When comparing the prevalence of myopia in children across different age groups, high school students had the highest prevalence, which may be related to the long hours of learning and limited outdoor activities during high school. Increased ultraviolet radiation might shape health-related behaviors. Greater sunlight exposure had been linked to increased outdoor activity and a reduced risk of myopia, although environmental constraints such as cold temperatures and limited recreational facilities might restrict physical activity patterns.^[26]^ The high prevalence of myopia can have a long-term negative impact on students’ academic performance and mental health, requiring special attention.

A previous study of 504 primary school students in Lhasa, the capital of Tibet, reported a dental caries prevalence of 79.76%.^[27]^ Another study showed an 87.6% prevalence of dental caries in 298 Tibetan children aged 9 to 12 years living in Gansu, a low-altitude region near Tibet in China.^[28]^ A third study in India found that 79.5% of 254 Tibetan children aged 6 to 18 years had dental caries.^[29]^ In the current study, the prevalence of caries among 7- to 18-year-old children and adolescents was 70.3%. Although the prevalence of dental caries in Tibetan primary and secondary school students was lower than previously reported, two-thirds of students still had dental caries, indicating a widespread health issue in Tibetan areas. The high prevalence of dental caries not only affects students’ diet and nutrient absorption but may also lead to other oral diseases, ultimately impacting overall health. Toothbrushing, routine dental visits, and daily vegetable intake were important factors associated with the incidence of dental caries. It was reported that only 22% of 232 Tibetan students in Shigatse brushed their teeth in the morning and evening. Several factors could contribute to this pattern. First, access to dental health services in remote areas such as Shigatse might be limited, as a previous study in Tibetan populations reported low treatment and filling rates, indicating insufficient availability of oral healthcare resources.^[30]^ Second, oral health awareness and preventive practices might be relatively inadequate in rural communities, where poor hygiene behaviors (e.g., infrequent tooth brushing) have been identified as significant risk factors for dental caries.^[31]^ Finally, local dietary patterns might also contribute to the high prevalence. Diets characterized by high carbohydrate intake and limited access to fresh foods have been associated with increased caries risk.^[32]^

Due to the special environment of the plateau region of Tibet, the physical development of children and adolescents generally lags behind that of their peers in lowland areas.^[33]^ The average height and weight of school-age children in Tibet are lower than the national averages.^[34]^ The prevalences of underweight, obesity, overweight, and growth retardation were 19.6%, 1.1%, 3.9%, and 7.6%, respectively, in Tibet, compared with 8.0%, 18.1%, 5.2%, and 19.7% among Tibetan and Mongol children and adolescents in Golmud, Qinghai, China, in 2022.^[35]^ Compared with ethnic minorities in Qinghai, Tibetan children and adolescents had a lower prevalence of obesity, but underweight and growth retardation were more common. The differing prevalences of obesity among Mongolian and Tibetan children may be due to dietary differences.

**Figure 4.**
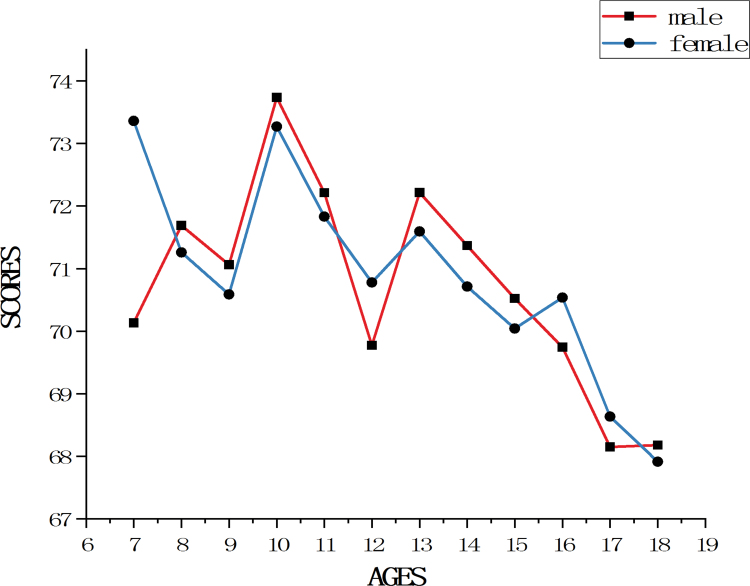
Trend of height, weight, and lung vital capacity changes of primary and middle school students of different sexes in different years (A, height in boys; B, height in girls; C, weight in boys; D, weight in girls; E, lung vital capacity in boys; F, lung vital capacity in girls). Data for 2014 and 2019 were from references 36 and 10, respectively.

The traditional Tibetan diet is dominated by rice, barley, vegetables, and dairy products, with a relatively low intake of meat, whereas the diet of Mongols is high in meat and dairy products and correspondingly high in energy and fat. Diets in rural Tibetan areas are often characterized by limited diversity, which, combined with geographic isolation and restricted access to healthcare, might increase the risk of nutritional deficiencies and growth retardation.^[18]^ The height of Tibetan primary and secondary school students changed with age. Before age 13, the average height and weight of boys and girls in low-altitude areas were higher than those in high-altitude areas. Compared with the scientific reference values for the growth and development of Tibetan children developed by Ma et al,^[36]^ our study found that more than 55% of the children and adolescents had better height, weight, and vital capacity measurements than those in the reference standards, although they were still lower than the national physical health standard for Chinese students. Considering our results and previous findings on the average height and weight of Tibetan primary and secondary school students in 2014 and 2019, published by the Chinese Student Physique and Health Research Group,^[6,37]^ differences in height and weight by sex from 2014 to 2024 were statistically significant (*P* < .05). The height and weight of students by sex changed with age, as shown in Figures 4A–D. Based on previous studies showing that vitamin D deficiency is associated with growth retardation in children, an analysis of vitamin D nutritional status in Tibetan children aged 0 to 12 years found that vitamin D deficiency is common among Tibetan children.^[38]^ Any form of malnutrition can seriously threaten children’s health. Short-term harms are chiefly manifested as physical and mental retardation, and long-term harms include cognitive impairment, reduced learning and working capacity, and increased risks of cardiovascular disease, diabetes, hypertension, and other chronic conditions. The dietary habits of Tibetan children and adolescents need improvement. Regular vitamin D supplementation should be implemented in schools. Foods rich in vitamin D should be provided to meet the requirements of children and adolescents. Outdoor activities: for example, incorporating dances into routine exercises can be encouraged to appropriately increase skin exposure to sunlight.

Children and adolescents living at high-altitude are exposed to environmental conditions that may influence growth and health. Chronic hypoxia, a defining feature of plateau regions such as Tibet, can affect cardiopulmonary function and physical development, and has been associated with slower growth and lower height and weight compared with low-altitude populations.^[19]^ Hypoxia is a common issue at high altitude. The hypoxic environment can lead to declines in lung function and reduced gas-exchange efficiency.^[39]^ Prolonged hypoxia can delay physical development, including thoracic cavity development, in children and adolescents, resulting in low vital capacity. Available evidence suggests that Tibetan people are better adapted to high-altitude environments.^[40]^ However, hypoxia, combined with high-altitude climatic conditions, can limit the duration and types of outdoor sports in which students can participate, further constraining the development of lung function in children and adolescents. In a study comparing Tibetan children and adolescents in Lhasa between 1985 and 2019, vital capacity showed an increasing trend before puberty and a decreasing trend after about age 15.^[41]^

Considering our results together with the vital capacity data for Tibetan primary and secondary school students in 2014 and 2019 published by the Chinese Student Physique and Health Research Group,^[6,18]^ vital capacity from 2014 to 2024 differed significantly between sexes (*P* < .05). The trends in vital capacity by sex across ages are shown in Figures 4E and 4F. In the future, interventions should be implemented to improve the lung vital capacity of Tibetan students, ensuring normal physical and mental development.

ANN models are well suited for modeling complex nonlinear relationships among multiple predictors.^[42]^ In health assessment studies, anthropometric measurements, physiological functions, and health disorders may interact in multidimensional ways that are not fully captured by traditional linear models.^[43]^ Therefore, ANN models can provide improved predictive performance. In recent years, they have been increasingly applied in public health research, including studies on obesity risk and nutritional status in children and adolescents.^[44]^ When applying an ANN model to predict the comprehensive score, we also included altitude as an additional independent variable to improve the model’s predictive ability for health status.^[45]^

## 5. Conclusion

The overall health status of primary and secondary school students in Tibet was moderate. Many students had health issues, including myopia, dental caries, severe underweight, underweight, overweight, obesity, and growth retardation. There is an urgent need for improved health education, nutritional supplementation, and resource optimization to promote the physical health of children and adolescents on the Tibetan Plateau.

## Acknowledgments

We would like to appreciate all the Shigatse students and field organization teachers who participated in the data collection.

## Author contributions

**Data curation:** Chenyao Mai, Xiaofei Liu, Qiuyu Pan.

**Funding acquisition:** Chenyao Mai, Rulin Li, Pengcheng Zhang.

**Investigation:** Chenyao Mai, Pengcheng Zhang.

**Validation:** Qiuyu Pan.

**Conceptualization:** Rulin Li.

**Supervision:** Rulin Li.

**Project administration:** Jun Hu.

**Writing – original draft:** Chenyao Mai.
